# Children's Ability to Identify and Describe Dysphonia: A Multimethod Study

**DOI:** 10.1111/1460-6984.70197

**Published:** 2026-01-21

**Authors:** Carlotta De Biasio, Ciarán Kenny

**Affiliations:** ^1^ Department of Clinical Speech and Language Studies Trinity College Dublin Dublin Ireland

**Keywords:** awareness, children, paediatric dysphonia, voice disorders

## Abstract

**Background:**

Engaging children with voice therapy is a commonly reported clinical challenge. It is unknown whether this is because children cannot always perceive dysphonia, or whether clinicians do not explain it in age‐appropriate terms.

**Aims:**

This research aimed to analyse whether children can identify the presence of dysphonia in other children's voices and understand how they describe dysphonic and non‐dysphonic voices.

**Methods and Procedures:**

The research was carried out using a multimethod design combining quantitative and qualitative approaches. Thirty children aged 6;4–10;6 (17 male, 13 female) listened to 5 dysphonic and 5 non‐dysphonic paediatric voice samples. They were asked whether the voices sounded ‘OK’ or ‘Not‐OK’. The relationships between sex, age, and the percentage of correct responses were analysed using the Mann–Whitney *U* test and Spearman's correlation. Participants were asked to describe one non‐dysphonic and one dysphonic voice sample in their own words. Descriptions were analysed using content analysis to thematically categorise responses.

**Results:**

Median correct answers for identifying dysphonia presence were 70% (IQR = 20). No statistically significant difference between sexes was found (*p* = 0.432, power = 0.53). Results showed no correlation between age and percentage of correct answers (*p* = 0.751, power = 0.83). Content analysis identified three categories: (a) *Words describing voices*, featuring adjectives related to voice quality, (b) *Extra information*, concerning speech and voice but not voice quality, (c) *Unrelated information*, irrelevant to communication.

**Conclusions and Implications:**

Children are capable of correctly identifying dysphonia presence in other children's voices with a high degree of accuracy, unrelated to their sex or age. However, the lack of universal appreciation of dysphonia means that clinicians should examine children's perceptual abilities before commencing voice therapy. Children sometimes mistake voice quality for other concepts, both communication‐related and not. The findings from this study provide age‐appropriate descriptive terms that could facilitate education of paediatric clients about the concept of voice quality.

**WHAT THIS PAPER ADDS:**

*What is already known on the subject*

- There is little knowledge about age‐related understanding of paediatric dysphonia, and the barriers that weaken adherence to voice therapy are numerous, including children's awareness of their voice disorder and limited knowledge about how the voice is produced. Past studies have demonstrated that children with dysphonia are aware of their voice disorder and its impact on their daily lives. However, these findings were based on structured questionnaires that may have influenced the responses. We therefore wanted to investigate whether children can distinguish between dysphonic and non‐dysphonic voices and understand how they describe dysphonic and non‐dysphonic voices when asked an open‐ended question.

*What this study adds to existing knowledge*

- The results of the present study demonstrate that children can identify the presence of dysphonia in other children's voices with 70% accuracy. Accuracy is unaffected by sex or age. Children can express themselves about how voices sound, and the language is extremely varied, but they tend to mistake voice for other concepts.

*What are the potential or actual clinical implications of this work?*

- Since children cannot consistently perceive dysphonia with absolute accuracy, improving their ability by training them, and consequently enhancing their awareness of the voice disorder, can potentially benefit voice therapy. Similarly, if clinicians teach children what voice and dysphonia are by using age‐appropriate descriptive terms, some of which are suggested in the present findings, compliance, therapy adherence and consequently therapy efficacy could be positively impacted.

## 1 Introduction

Children with dysphonia are usually aware of their voice disorder and its impact on their daily lives (Connor et al. 2008; Verduyckt et al. 2011). Research involving children and adolescents between the ages of 2 and 18 (Connor et al. 2008) and between 5 and 13 (Verduyckt et al. 2011) highlighted concerns across the physical, social/functional and emotional domains, indicating a broad awareness of the condition's effects (Connor et al. 2008; Verduyckt et al. 2011). It has been demonstrated that dysphonic children are judged more negatively than non‐dysphonic ones on attributes unrelated to voice, such as appearance and personality, by listeners of different ages and backgrounds (Ruscello et al. 1988; Lass, Ruscello, Stout, and Hoffmann 1991; Lass, Ruscello, Bradshaw, and Blankenship 1991; Ma and Yu 2013).

Since the majority of children with dysphonia are aware of their condition and recognise its impact, voice therapy plays a crucial role in helping children obtain a healthy voice. Recent studies have demonstrated that voice therapy yields positive outcomes for children with dysphonia (Trani et al. 2007; Tezcaner et al. 2009; Valadez et al. 2012; Akin Şenkal and Çiyiltepe 2013). However, adherence to therapy is often suboptimal, which can hinder the achievement of treatment goals (Mori 1999; Brehm et al. 2018). Adherence to paediatric voice therapy is fundamental to achieving therapeutic goals and understanding the barriers that clients encounter can help clinicians and patients ensure success (Braden et al. 2018). Barriers to adherence include a lack of awareness of vocal symptoms, which can affect a child's motivation to participate in therapy (Mori 1999), as well as parental beliefs that children may not fully understand the therapeutic process (Braden et al. 2018). Indeed, some children report limited knowledge of voice production and how voice therapy works, often citing abnormal voice quality as a motivator for seeking treatment (Braden et al. 2018).

While previous studies have shown that children are aware of their dysphonia (Connor et al. 2008; Verduyckt et al. 2011), these findings were based on structured questionnaires that may have influenced the responses. There is a need to quantify how accurately children perceive dysphonia and to explore their perspectives using open‐ended questions, which could offer more natural insights into how they understand their voice disorder. This research aims to address these gaps by evaluating children's ability to identify dysphonia in other children's voices and by exploring the vocabulary they use to describe both dysphonic and typical voices. Since sex differences in subcortical auditory processing have been shown to emerge across development (Krizman et al. 2019), sex was included as an exploratory variable in the present study. The findings from this study could provide valuable insights for clinicians treating children with dysphonia, offering information on children's general awareness of the condition and an age‐appropriate vocabulary to facilitate discussions about voice disorders.

### 1.1 Aims and Objectives

The aim of this research is to enhance our understanding of how children perceive and describe dysphonic and non‐dysphonic voices. Specifically, the study seeks to: (a) assess children's ability to distinguish between dysphonic and non‐dysphonic voice samples and (b) explore the descriptive language children use when talking about voice.

## 2 Material and Methods

### 2.1 Study Design

This study employed a multi‐method design. Two distinct tasks were carried out in parallel to address the two research objectives. Unlike mixed‐methods research, multi‐method designs allow researchers to investigate independent yet complementary questions (Morse 2003).

The author's Institutional Review Board approved the study (24.04.2024). Parents were provided with an information leaflet outlining the study and its inclusion and exclusion criteria. They were asked to confirm that their child met these criteria when providing written consent. Parents were likewise given an equivalent child‐friendly version and asked to discuss study participation with their child. Children who returned a signed consent form were invited to participate. Eligibility was monitored by the researchers at the point of data collection, and children who did not meet the inclusion criteria were excluded. Written informed consent was obtained from the participants’ parents, and written informed assent was obtained from the participants.

### 2.2 Participants

Convenience sampling recruited children who met the following inclusion criteria: (1) aged 5 years 0 months to 10 years 11 months, (2) proficient English speakers, (3) no intellectual disability, (4) normal hearing range (with or without the use of a hearing aid). A total of 32 children were recruited, but one child withdrew. Another child was referred to the study by their parent but was deemed ineligible to participate, as he could neither speak nor understand English to the level required by the inclusion criteria. Participant characteristics are in Table 1.

**TABLE 1:** Participant characteristics.

		Total	Males	Females
**Number of participants**		30 (100%)	17 (56.7%)	13 (43.3%)
**Median age (IQR)**		8;4 (Q1 6;11–Q3 9;9; IQR 2;10)	8;7 (Q1 6;10–Q3 9;10; IQR 3;0)	8;2 (Q1 7;6–Q3 9;1; IQR 1;7)
**Age range**		6;4–10;6	6;4–10;4	6;5–10;6
**Key age groups**	**6;4–6;11**	9	6	3
	**7;0–7;11**	2	0	2
	**8;0–8;11**	8	4	4
	**9;0–9;11**	5	3	2
	**10;0–10;6**	6	4	2

*Note*: Ages reported as years;months.

Participants’ voices were evaluated independently by both researchers using the overall grade of hoarseness (G) from the GRBAS scale (Hirano 1981). Agreement in scores occurred for all but one participant, which was resolved through consensus discussion. Two children (6.7%) had mild dysphonia, one (3.3%) had moderate, and none had severe. All remaining children (*n* = 27/30; 90%) had no dysphonia.

### 2.3 Instruments and Materials

Ten voice samples for use as stimulus items were obtained from five dysphonic and five non‐dysphonic children following parental consent. The voice samples consisted of connected speech and were recorded in a quiet environment. For each sample, children were asked to describe their favourite hobbies. Samples were approximately 10 s long and were recorded using a digital voice recorder (OM SYSTEM WS‐828). Spectrographic analysis and harmonics‐to‐noise (HNR) ratio were investigated for all samples to eliminate background noise as a confounder. Spectrogram confirmed the absence of background noise. Dysphonic samples showed lower HNR values (*M* = 8.12 dB, SD = 3.4) than non‐dysphonic samples (*M* = 12.73 dB, SD = 3.1), consistent with intrinsic phonatory noise rather than environmental interference. Both investigators blindly and independently rated the voice quality of the dysphonic children used as stimulus items, reaching agreement on their overall grade (G) of dysphonia on GRBAS (Hirano 1981) through consensus. Of the five dysphonic children, one had mild dysphonia, two moderate, and two severe.

To address the first objective (assessing children's ability to distinguish between dysphonic and non‐dysphonic voices), participants completed a categorisation task. They listened to all 10 voice samples and indicated whether each voice sounded ‘OK’ (typical) or ‘Not‐OK’ (dysphonic). To facilitate responses, participants were provided with thumbs‐up and thumbs‐down paddles, which they could use if they wished. The order of voice sample presentation was randomised using a computer‐generated true randomisation sequence.

To address the second objective (exploring the descriptive language children use to characterise voices), a spontaneous description task was conducted. Participants were asked to describe one dysphonic and one non‐dysphonic voice. Samples were selected through computer randomisation, resulting in children listening to different pairs of samples from one another. Before completing the task, they were first shown a stuffed teddy bear and provided with a verbal description of the toy (Appendix A). This was to ensure the understanding of the concept of ‘describe’ but by using a visual stimulus unrelated to voice, so as not to influence their responses.

All voice samples were played through Logitech MX Sound speakers positioned on the table in front of participants. Participants’ descriptions of non‐dysphonic and dysphonic voice samples for the second task were recorded using a digital voice recorder (OM SYSTEM WS‐828).

### 2.4 Procedures

Data collection was conducted in person in a quiet room within a primary school that agreed to facilitate recruitment. Before commencing the tasks, the researcher spent a brief period engaging the child in informal conversation (e.g., introducing themselves and asking how the child was) to build rapport and ensure the child felt comfortable. The study procedures were then explained in age‐appropriate language, and the child was asked whether they were happy to take part before beginning. Written assent was then obtained at the start of the session to reconfirm willingness to participate.

For the first task, the researchers played the randomly ordered non‐dysphonic and dysphonic voice samples once. Following each track, participants were asked whether the sound of the voice from the recording was ‘OK’ or ‘Not‐OK’. These terms were chosen because the only salient difference between recordings (other than speaker characteristics like accent and sex) was the presence/absence of dysphonia. Alternative labels (e.g., good/bad, healthy/unhealthy) were rejected, given their potential to bias findings from the second task. The number of correct responses was recorded. A training set was not used since the study's purpose was to identify whether children could identify dysphonia without any cueing.

When the first task concluded, children listened to the description of the teddy bear (Appendix A). The researchers explained that participants would hear two voice samples and that they would be asked to describe those voices using the words that came to their mind while listening to the audio. Following each voice sample, participants were asked, ‘How would you describe the voice you just heard? What words came to your mind when you heard that voice?’ Their responses were recorded using a digital voice recorder and later transcribed verbatim.

During data collection, fieldnotes were taken with observations about participants’ behaviours and comments. Given their young age, this was to identify whether participants had potential difficulties attending to or understanding tasks.

### 2.5 Data Analysis

For the first task, the dependent variable of interest was the percentage of correct responses to the presence of dysphonia in stimulus voice samples. Independent variables were participants’ age and sex. During data collection, fieldnotes indicated that some children may not have fully understood tasks. Fieldnotes collected by the researcher showed that such children talked about liking or disliking the voices. An additional independent variable was created to determine whether there were significant differences in results between those suspected of reduced understanding versus those with good understanding.

Sample size estimation was conducted using G*Power 3.1 software (Faul et al. 2007). Using conventional clinical research settings (Wang and Ji 2020), power was set at 0.8, with a large effect size and *α* = 0.05. The highest sample size requirement (n = 52) was taken for the study. The study failed to recruit sufficient participants from the study site. Post‐hoc power analysis was therefore carried out, where statistical testing failed to reach sample size requirements.

Descriptive and inferential data analysis were conducted with IBM SPSS Statistics version 28.0 (IBM Corporation, Armonk). Age was measured in months for greater precision of analysis and was non‐normally distributed, so reported using median (IQR). Participants’ percentage of correct guesses was non‐normally distributed.

For inferential analysis, test assumptions were met unless stated otherwise. Mann–Whitney *U* test was used to analyse the influence of sex on the correct identification of dysphonia from stimulus items. Spearman's correlation determined the association between participants’ age and the number of correct responses. Ordinal logistic regression analysed the influence of sex and age on the percentage of correct responses. The dependent variable represented the proportion of correct responses out of 10 stimuli and therefore took only discrete ordered values in 10% increments. Because these scores did not behave as continuous ratio data and several lower categories were unoccupied, the outcome was treated as an ordinal variable rather than analysed with a linear model. An ordinal regression model was therefore estimated to explore the potential influence of age and sex. The proportional‐odds assumption was assessed using the Test of Parallel Lines and was violated (*p* < 0.001), indicating that the effect of the predictors was not constant across outcome thresholds; the results of the ordinal model are therefore interpreted with caution.

Qualitative data were analysed with conventional content analysis (Hsieh and Shannon 2005), where categories and codes were developed inductively from the data without influence from existing literature. All audio recordings were transcribed verbatim by the first author, who re‐listened to the recordings during coding to check transcription accuracy. The first author generated codes through repeated reading of the transcripts, comparing different coding structures to ensure that the coding remained grounded in participants’ own wording, with reflexive consideration of interpretation throughout the analytic process. In instances where the meaning of a child's response was unclear, a second author listened to the original audio, and discussion was used to confirm the interpretation. Codes were then organised into categories and subcategories through an iterative, data‐driven process.

## 3 Results

### 3.1 Children's Ability to Identify Dysphonia

Participants had a median of 70% (IQR = 20, range = 50–100) correct answers. This was the same in males and females (Mdn = 70), but females showed a higher IQR and wider range (IQR = 15, range = 50–100) compared to males (IQR = 11.7, range = 50–90). No statistically significant difference between the two sexes in their ability to correctly guess the presence of dysphonia in the voice samples was identified (*U* = 91.0, *p* = 0.432, power = 0.53). A negligible, non‐significant correlation (*r_s_* (28) = –0.060, *p* = 0.751, power = 0.83) between participants’ age and the number of correct responses to the presence of dysphonia in the voice samples was identified.

The ordinal model fitting information table showed *p* = 0.644, indicating that the model did not fit the data well. Significance values for both the Pearson (*p* = 0.063) and Deviance (*p* = 1.000) Chi‐square Goodness of Fit tests were not statistically significant. Cox and Snell *R* ^2^ was 0.029, suggesting that the data do not fit the regression model adequately. Neither the factor ‘sex’ (*p* = 0.363) nor the covariate ‘age’ (*p* = 0.744) was found to significantly contribute to the model, indicating that factors other than these are responsible for the number of correct or incorrect guesses.

When answering the question about whether the voices sounded ‘OK’ or ‘Not‐OK’, three children were observed to have potential difficulties understanding the task. A brief sensitivity check was conducted to assess whether their inclusion materially altered the results. Children who appeared to understand the task had a median of 72.1% correct responses (IQR 20, range 50–100), while children with potential misunderstanding had a median of 66.7% (IQR 0, range 60–70). Mann–Whitney *U* identified no statistically significant difference in scores between the participants who appeared to understand the task and those who may have had difficulties (*U* = 30.5, *p* = 0.509, power = 0.23), suggesting that retaining these children did not affect the overall findings. All participants were therefore included in the primary analysis.

### 3.2 Descriptions of Voices

Since the anticipated sample size was not reached, random selection of one dysphonic and one non‐dysphonic voice sample for judgement meant that not all stimulus voice samples were played an equal number of times for this task. The voices with a mild overall grade of dysphonia were played 5 times, moderate dysphonia 14 times, and severe dysphonia 11 times.

Content analysis generated three categories: *Words describing voices*, *Extra information* and *Unrelated information* (Table 2). The category *Words describing voices* comprises all the information that accurately describes voice quality. The category *Extra information* includes details related to speech and voice, but that do not pertain directly to voice quality. *Unrelated information* concerns information irrelevant to the task.

**TABLE 2:** Content analysis of the description of voices.

Categories	Sub‐categories	Codes (no. participants reporting code)			
		Dysphonic	Example quotes	Non‐dysphonic	Example quotes
Words describing voices	Adjectives related to voice	Loudness (3)	P14: It gets louder and louder and quieter and quieter and then louder and louder and quieter and quieter. P20: Like if you're whispering basically.	Loudness (3)	P1: It's quiet. P7: It's a bit loud.
		Pitch (1)	P11: Deep.	Pitch (1)	P11: Not that deep.
		Quality (13)	P9: It was nice. P14: Probably a very dry it's a very dry voice, very dry. P23: The only word that comes to my mind is dehydrated because he sounds like he hasn't drank water in five hours. P23: I just think he sounded weird. P15: It's kind of scratchy. P27: His voice was a little bit cranky. P28: It was soft. P28: His voice was kind of wrinkly. P30: It was a voice crack.	Quality (8)	P9: It's really nice. P12: Weird again. P12: Low. P22: Like soft. Like kind of like very feminine. P26: Cute.
	Positive‐negative nouns	Good (3)	P2: Good. P10: It sounded ok.	Good (9)	P10: I'll say that it's OK. P14: It's like normal. P19: A good voice.
		Bad (4)	P3: It's like really bad. P5: It's not good.	Decent (1)	P9: Pretty decent.
	Sore throat	Being sick or having a sore throat (6)	P1: A sore neck. P6: Like kinda sick, like he's sick and he has like a sore throat.	Being sick or having a sore throat (2)	P1: Sort of a sore voice. P15: It feels like when somebody is sick.
	Age			Young (1)	P26: It's young.
Extra information	Audio content	Personal point of view on the audio content (4)	P6: (…) He probably won like a shirt or something and the bottoms of it like Messi or, you know, those shirts. I don't know which one. P9: I like the way that he's talking about self‐defence.	Personal point of view on the audio content (2)	P6: I would say that he's probably thinking about, so I don't think he's talking about like when he's going to bed or something like he's probably angry. (…)
		Reporting the audio content (5)	P27: He was talking about his football match. P28: I think they were talking about someone.	Reporting the audio content (6)	P24: Oh, it sounds like a kid explaining to his auntie about I'm going to the trip to a country. (…)
	Components of speech	Fluency (1)	P7: Like a little bit stuttery.	Fluency (1)	P7: Not that much stuttering.
		Accent (2)	P19: It sounds like a posh accent.	Accent (1)	P14: The voice sounds like German slash Italian.
				Articulation (1)	P21: She speaks clearly.
	Emotions and feelings	Sad or calm or kind (2)	P2: Sad. P21: Calm.	Sad or calm or kind (4)	P8: Sounds very kind, you know. P27: She had a really calm voice.
				Funny or happy (2)	P20: Like someone like if you're happy and like you're excited to do something.
	Scoring			Score (1)	P15: Let's give it a 70 out of 100.
Unrelated information	Comparisons	Similar voice (1)	P4: Like somebody from 3rd class.	Similar voice (2)	P8: Kinda like mine actually.
	Liking/Disliking			Liking the voice (1)	P11: I like it.

**FIGURE 1:**
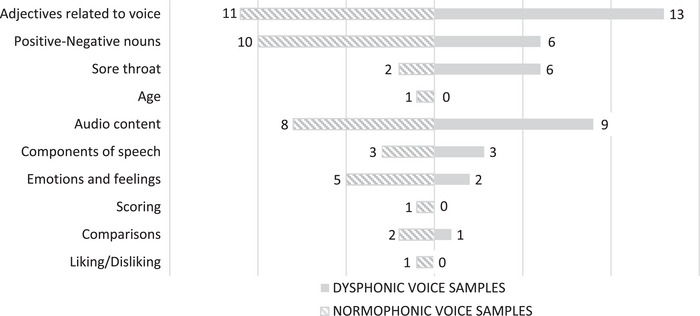
Frequency distribution of the subcategories among dysphonic and non‐dysphonic voice samples.

Table 3 reports the frequency distribution of the categories among the 30 participants listening to the voice samples. Figure 1 illustrates how the subcategories are distributed among the dysphonic and non‐dysphonic voice samples.

**TABLE 3:** Frequency distribution of the three categories among dysphonic and non‐dysphonic voice samples.

Category	No. of children using the category for the dysphonic voice sample (%)	No. of children using the category for the non‐dysphonic voice sample (%)
Words describing voices	25 (83.3%)	24 (80%)
Extra information	14 (46.7%)	17 (56.7%)
Unrelated information	1 (3%)	3 (10%)

## 4 Discussion

### 4.1 Children's Ability to Distinguish Between Dysphonic and Non‐Dysphonic Voice Samples

The findings from our study showed that children within our sample were able to identify the presence of dysphonia with an average of 70% accuracy. Children rarely achieved the highest score, and large differences in accuracy between individual participants were observed. These results could imply that, even with adequate auditory perception, they still face challenges in distinguishing between dysphonic and non‐dysphonic voices.

The present study's results partially align with those of Lass, Ruscello, Stout, and Hoffmann (1991), who demonstrated that children would judge dysphonic and non‐dysphonic voices differently when given a list of bipolar adjectives to use to rate the voices. This current study, however, provides an estimate of the extent to which children can identify the presence of dysphonia when given as little help as possible.

#### 4.1.1 Sex and Age

Current findings suggest no difference between males and females in their ability to identify dysphonic versus non‐dysphonic voices. One study found that females tend to maintain faster and more accurate auditory responses as they age compared to males (Krizman et al. 2019). These sex differences become more pronounced over time, being more evident in adults than in children. Krizman et al. (2020) reported that during adolescence and young adulthood, the decline in harmonic encoding was faster in males than in females. It may therefore be the case that the differences between males and females in their ability to identify the presence of dysphonia in other children's voices become more distinct during adolescence or early adulthood, meaning that dysphonia discrimination could differ in older children. It must be acknowledged that the current findings may represent a Type II error since sample size requirements were unmet. Challenges in rating dysphonia and its severity are known to exist even in adulthood. Judgments of dysphonia improve both with experience and when raters receive preparatory perceptual training (Benoy and Jayakumar 2024; Brinca et al. 2015). Since these studies were conducted with adult listeners, it would be interesting to investigate how such perceptual training may affect child listeners.

### 4.2 Children's Vocabulary When Describing Voices

When asked how they would describe the two voice samples, the majority of children did describe voice quality, suggesting that most children are aware of what voice is and can adequately discuss the concept. This is similar to findings from previous studies by Connor et al. (2008) and Verduyckt et al. (2011), which explored children's awareness of and perspectives on dysphonia by using a series of questions related to the voice disorder. In our study, however, children were given an open‐ended question that asked them to describe the voice without prompts.

The decision to use one open‐ended question created a diversity of answers, shedding light on how children conceptualise voice, while simultaneously highlighting their difficulty in doing so. Children confusing voice with other concepts, such as audio content or components of speech, is a sign that not all children have a clear understanding of what voice is. The answers given by the participants of this study are consistent with previous research (Verduyckt et al. 2012, 2011), which pointed out children's tendency to mistake voice for articulation or speech disorders. A small percentage of participants answered the question by providing information unrelated to voice or communication. No child asked the researcher to reformulate the question, but future studies could benefit from verifying children's understanding. This could be achieved by asking participants if they understood the task, or by having them reformulate what is being asked of them in their own words. Conversely, children in this study might have understood the task, but had limited vocabulary or knowledge of voice to answer.

During data analysis, the researcher identified recurring patterns in the concepts and words children used to describe the two groups of voices. In some instances, identical terms (e.g., ‘nice’) were used for both dysphonic and non‐dysphonic voices. Rather than being excluded, these responses are informative, as they suggest that children may not yet hold a differentiated conceptual framework for dysphonia or may lack the linguistic repertoire to express such distinctions. As a result, the same subcategories and codes were applied to both dysphonic and non‐dysphonic groups. This approach led to the creation of Figure 1, which compares the frequency distribution of subcategories across the two groups. The figure highlights how children often used the same attributes to label both dysphonic and non‐dysphonic voices. It also reveals how certain attributes are more frequently used to describe dysphonia (e.g., sore throat), and others to talk about non‐dysphonic voices (e.g., emotional adjectives). Future research could explore whether significant differences exist in the qualitative attributes children use to describe dysphonic versus non‐dysphonic voices.

### 4.3 Misunderstanding of the First Task

While children who potentially misunderstood the first task were few, it is nonetheless of interest to understand how and why the misunderstanding may have happened. Children were asked the question “Do you think this voice sounds OK or Not‐OK?”.N The answers of children who appeared to have misunderstood the question could be because of two factors: misinterpretation of the words ‘OK’ and ‘Not‐OK’, and misconception of what is meant by ‘voice’.

Previous studies investigating children's awareness of voice disorders indicate children's tendency to confuse voice with articulation, phonology and other speech features (Verduyckt et al. 2012, 2011). While this distinction could have been presented in advance to our study participants, this may have led to information bias and would not have represented a naïve attempt to rate voice quality. It could likewise have influenced responses to the second task by providing participants with specific communication‐related terms. Future studies could explicitly distinguish between voice and other speech features if this would not interfere with study outcomes.

### 4.4 Implications for Clinical Practice

Our findings could help to enhance children's compliance with voice therapy. By providing context about whether children can discriminate dysphonic from non‐dysphonic voices, clinicians and young clients may be better able to set therapy goals and understand whether therapeutic voice improvements are being made.

This could contribute towards a stronger therapeutic alliance, which refers to the collaborative relationship between the therapist and the client (Martin et al. 2000). Therapeutic alliance is considered key to successful therapy by clinicians and parents, more than by children (Braden et al. 2018). Improved awareness of their voice disorder could positively impact dysphonic children's motivation to adhere to voice therapy and consequently improve therapeutic outcomes.

### 4.5 Strengths and Limitations

To the best of the researcher's knowledge, the present study was the first to explore the ability of the paediatric population to recognise the presence of dysphonia in the voices of other children and to understand the vocabulary they use to talk about both dysphonic and non‐dysphonic voices. Consistent data collection procedures and validated scales were used to avoid measurement bias and enhance validity and reliability.

A limited number of stimulus items were used for both tasks to prevent children from becoming bored and providing spurious answers, thereby enhancing the results’ credibility. However, because each item within the first task was administered only once, it was not possible to examine the intra‐rater reliability of children's responses to the presence or absence of dysphonia. Future research could consider investigating how reliable children's responses are over time.

To limit response bias, the researchers avoided the use of voice‐related terminology with participants during data collection and utilised an open‐ended question when asking participants to describe audio samples. The decision to use the adjectives ‘OK’ and ‘Not‐OK’ was made to avoid the risk of associating positive and negative emotions with non‐dysphonic and dysphonic voices, respectively, and therefore avoid emotional bias. However, the choice of labelling the two groups of voices might have influenced children, as some of them used those same labels in the second task. A solution may be to reverse the order of task administration in the future.

The choice of using a tangible object as an example of what a *description* is, instead of something perceptible by hearing, was made to avoid influencing children to use certain words commonly related to sounds. At the same time, it is possible that the description of the teddy bear has not achieved its purpose, because describing a physical object differs from describing the concept of voice.

For task two, the a priori sample size calculation allowed each dysphonic and non‐dysphonic stimulus item to be played an equal number of times. Since the target sample size was not reached, stimulus items were played an unequal number of times, potentially introducing a bias. The stimulus items were based on spontaneous speech samples (discussing hobbies) rather than a reading task to ensure that samples could be obtained from children without developed literacy skills. This naturally led to variation in speech characteristics and content that may have influenced results.

Selection bias could not be controlled due to limited engagement from local schools. All participants were drawn from a single school in a relatively low‐socioeconomic area. A proportion of participants were first‐ or second‐generation immigrants, which may influence sociocultural and sociolinguistic concepts of (ab)normal voice and the language used to describe it. Findings may therefore differ in samples drawn from other demographic or educational contexts. The recruited sample size did not reach the calculated requirements for each test, resulting in a decreased statistical power, though post‐hoc power analyses were provided for interpretation.

Observer bias and the Hawthorne Effect were unavoidable for this study. Contamination bias might have occurred because data collection took place in a school setting over a period of days. Participants whose data had not yet been collected might have been exposed to elements of the research by discussing with other participants about the research itself, inadvertently influencing the results.

### 4.6 Future Research

Future research should replicate the study with a larger sample size, which could also investigate differences in accuracy among key age groups. Future studies using balanced stimulus sets could also examine whether children's ability to identify and describe dysphonia varies systematically with dysphonia severity. Recruiting children from diverse backgrounds could help improve generalisability. Children in this study were not asked if they had ever seen a voice specialist, which could have influenced responses. A future study using dysphonic children may be helpful, since the acknowledged presence of a voice disorder could influence dysphonia awareness and conceptualisation, as well as communication‐related vocabulary. Future work using a larger set of stimulus voices may yield sufficient variability to support more robust regression modelling, allowing clearer examination of whether age or sex contributes to children's ability to identify dysphonia.

## 5 Conclusions

The findings of the present study suggest that children were partially aware of what voice and dysphonia are. This study demonstrated that most children were capable of correctly identifying dysphonia in other children's voices, but their accuracy varied from child to child, and neither age nor sex was associated with their accuracy.

Children can express themselves about how voices sound. It is crucial not to underestimate children's ability in this regard, but simultaneously not to assume their knowledge, since results from this study highlighted children's tendency to mistake voice for other concepts.

Given that children cannot consistently identify dysphonia with absolute accuracy, training them to accurately identify and describe it in advance of voice therapy could improve therapeutic alliance and treatment outcomes. Such training should use age‐appropriate descriptive terms. The present study provides an initial lexicon of terms used by children; however, clinicians are advised to confirm which descriptors resonate with each child, and to avoid using terms that children tend to apply to both dysphonic and non‐dysphonic voices.

## Ethics Statement

The author's Institutional Review Board approved the study (24.04.2024).

## Consent

Written informed consent was obtained from the participants’ parents and written informed assent was obtained from the participants. The audio samples are not publicly available due to privacy restrictions. Permission was not required to reproduce any materials within this manuscript.

## Conflicts of Interest

The authors declare no conflicts of interest.

## Data Availability

The data that support the findings of this study are available on request from the corresponding author. The data are not publicly available due to privacy or ethical restrictions.

## Appendix A Teddy Bear and Description of It Written By the Researcher

### A.1

‘The teddy bear is brown and fuzzy. It has black eyes, a dark brown nose and long arms. I think it's adorable, cute and sweet. It's probably soft and cuddly. I would love a teddy bear like this because you can hug it whenever you need to’.
